# Experience, Perceptions, and Barriers to the Management and Referral of Anorectal Disorders in Primary Healthcare: A Cross-Sectional Study in an Extreme Region of Chile

**DOI:** 10.7759/cureus.115024

**Published:** 2026-08-23

**Authors:** Francisco Navarro Subiabre, Tamara Contreras Ayca, Bruno Baluarte Llerena

**Affiliations:** 1 Surgery, Dr. Juan Noé Crevani Hospital, University of Tarapacá, Arica, CHL; 2 General Practice, Arica and Parinacota Health Service, Arica, CHL

**Keywords:** anorectal disorders, diagnostic accuracy, primary care physicians, primary health care, referral system

## Abstract

Background

Anorectal conditions are commonly managed in primary healthcare (PHC), but little is known about physicians’ experiences, confidence, and perceived barriers in geographically remote settings. Identifying these factors may help guide educational and healthcare-network improvement strategies.

Objective

To describe the perceptions, barriers, and experiences of physicians in the Destination and Training Stage (Etapa de Destinación y Formación (EDF)) working in PHC in an extreme region of Chile regarding the evaluation, management, and referral of anorectal disorders.

Methods

A descriptive cross-sectional study was conducted between August and September 2025 using an anonymous, self-administered 16-item questionnaire. All 54 EDF physicians working in PHC in the region were invited to participate. Categorical variables, including responses to Likert-type items, were summarized using absolute frequencies and percentages. Associations between selected ordinal variables were assessed using Spearman’s rank correlation coefficient.

Results

A total of 28 out of 54 physicians responded (51.9%). The most frequently reported conditions were hemorrhoids (56%) and anal fissure (29%). A moderate correlation was observed between perceived undergraduate training and confidence in performing the physical examination (ρ = 0.563; p = 0.002). Perceived functioning of the referral system was significantly associated with feedback from secondary care (ρ = 0.462; p = 0.013). The main barriers identified were lack of supplies, insufficient training, and absence of clear referral protocols.

Conclusion

Most EDF physicians reported encountering anorectal conditions, with predominantly educational and organizational barriers. Perceived training and coordination with secondary care emerge as key factors to improve the effectiveness and efficiency of management in PHC.

## Introduction

Anorectal disorders comprise a heterogeneous group of conditions affecting the anal canal and perianal region. In this study, the term refers primarily to common benign conditions encountered in primary healthcare (PHC), including hemorrhoidal disease, anal fissures, fistulas, perianal abscesses, and pilonidal disease. These disorders can cause bleeding, pain, pruritus, discharge, and alterations in defecation, with potentially substantial effects on quality of life and daily activities [1,2]. Their epidemiology is difficult to establish because definitions and diagnostic methods vary across studies, and many patients do not seek medical care. Hemorrhoidal disease, the most extensively studied condition, has an estimated global point prevalence of 25.9%, although substantial heterogeneity exists among populations and assessment methods [3].

The diagnosis of these conditions is primarily clinical and relies on a thorough medical history and careful physical examination. Initial management can generally be undertaken with non-surgical treatments that are within the scope of practice of any general physician. However, significant diagnostic inaccuracies have been reported in this group of conditions, potentially delaying appropriate treatment initiation and resulting in unnecessary referrals, with a consequent increase in healthcare costs [4].

In the Chilean public healthcare system, PHC is the principal entry point to the healthcare network. Physicians in the Destination and Training Stage (Etapa de Destinación y Formación (EDF)) program are recently graduated general physicians assigned to public healthcare facilities in vulnerable, rural, or geographically remote communities before entering specialist training [5]. Their clinical responsibilities include the initial evaluation, management, and referral of a broad range of conditions, often in settings with limited access to specialist care. However, little is known about EDF physicians’ training, confidence, clinical practices, available resources, or experiences with referral and counter-referral in the management of anorectal conditions.

Accordingly, this study aimed to describe the experiences, perceptions, and barriers reported by EDF physicians in the Arica and Parinacota Region regarding the evaluation, initial management, and referral of common anorectal conditions in PHC settings.

## Materials and methods

A descriptive cross-sectional study was conducted between August and September 2025 using an anonymous, self-administered survey administered to all physicians enrolled in the EDF program and working in PHC centers in the Arica and Parinacota Region, Chile.

The study population consisted of all physicians enrolled in the EDF program who were actively working in PHC facilities under the Arica Health Service during 2025. Given that the target population was limited and well-defined, a census sampling strategy was employed, aiming to invite all eligible physicians rather than calculating a probabilistic sample size. Therefore, no formal sample size calculation was performed. The final sample consisted of physicians who voluntarily completed the anonymous survey during the data collection period.

Inclusion criteria were (1) physicians enrolled in the EDF program, (2) active clinical practice in PHC centers within the Arica and Parinacota Region during the study period, and (3) voluntary acceptance to participate through informed consent. Physicians not working in PHC facilities, those outside the EDF program, and incomplete survey responses were excluded from the analysis.

**Figure 1:**
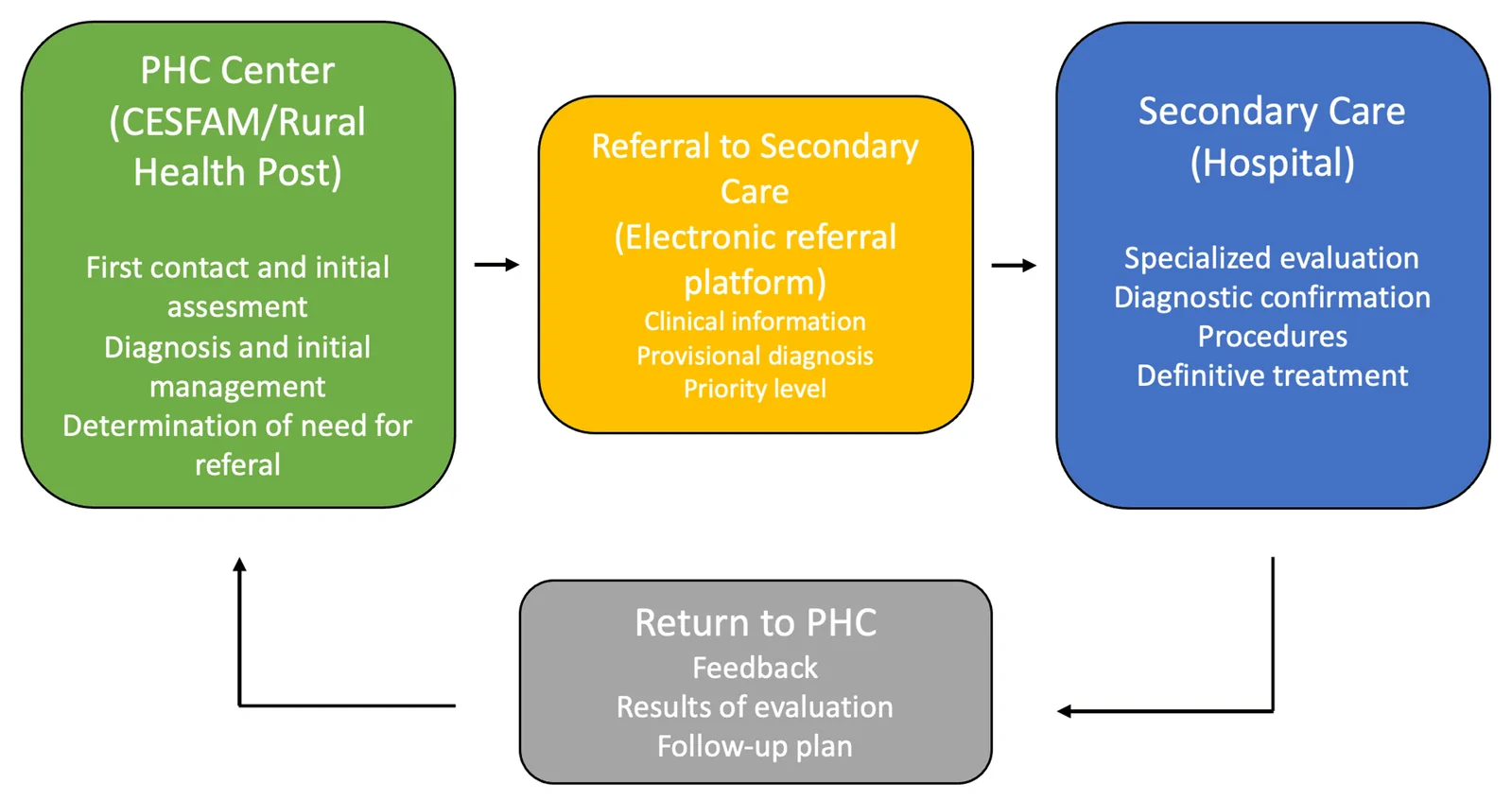
Chilean healthcare referral pathway from primary healthcare (PHC) to secondary care and the feedback loop for continuity of care CESFAM: Family Health Centers The image was created by the authors using Microsoft PowerPoint (Microsoft Corp., Redmond, WA, USA).

For contextual purposes, Figure 1 illustrates the referral pathway within the Chilean healthcare system, including the interaction between PHC and secondary care and the expected feedback process after specialist evaluation.

A structured 16-item questionnaire, divided into four sections, was developed by the authors based on a literature review and clinical experience. The items included in the survey were specifically developed for this study to assess physicians’ perceptions, confidence, and perceived barriers regarding the management of anorectal disorders in PHC (Table 1). Before its administration, the questionnaire was preliminarily evaluated by two PHC physicians from different healthcare centers in Arica to assess the clarity and comprehensibility of the items and the feasibility of completion. However, it did not undergo formal expert review or psychometric evaluation of validity or reliability.

**Table 1:** Questionnaire used to assess experiences, perceptions, and barriers regarding the management and referral of anorectal disorders among physicians working in primary healthcare The survey consisted of 16 items divided into four sections: employment information, clinical experience, diagnosis and referral practices, and perceived barriers and training needs. Likert-type items were scored using five-point response options.

Employment Information
What is your municipality of work? (Multiple choice)
In what type of healthcare facility do you work? (Family Health Center/Community Health Center/Rural Health Post)
How many years have you been working in primary healthcare? (<1; 1-3; 4-6; >6)
Clinical Experience
In the past month, how many patients with proctologic complaints or anorectal pathology have you treated? (0; 1-5; 6-10; >10)
What are the most frequent proctologic conditions in your clinical practice? (Multiple choice)
Hemorrhoids
Anal fissure
Anorectal abscess/fistula
Pilonidal cyst/abscess
Other (free text)
Diagnosis, Initial Management, and Referral
I feel confident and adequately trained to perform a proper proctologic physical examination when indicated. 1 = Strongly disagree 2 = Disagree 3 = Neutral 4 = Agree 5 = Strongly agree
I have adequate supplies and conditions to perform such examination (gloves, lighting, privacy). 1 = Strongly disagree 2 = Disagree 3 = Neutral 4 = Agree 5 = Strongly agree
I have access to basic initial treatments for the management of proctologic conditions in primary healthcare. 1 = Strongly disagree 2 = Disagree 3 = Neutral 4 = Agree 5 = Strongly agree
I have performed minor procedures related to proctologic conditions in PHC. Yes/No
I am familiar with the criteria for referring proctologic conditions to secondary care. 1 = Strongly disagree 2 = Disagree 3 = Neutral 4 = Agree 5 = Strongly agree
The referral/consultation system functions in a timely manner. 1 = Strongly disagree 2 = Disagree 3 = Neutral 4 = Agree 5 = Strongly agree
I receive feedback for most of the cases referred. 1 = Strongly disagree 2 = Disagree 3 = Neutral 4 = Agree 5 = Strongly agree
Barriers and Needs
What are the main barriers to the management or referral of proctologic conditions in primary healthcare? (Multiple choice)
Lack of training or knowledge
Lack of supplies/equipment
Infrastructure problems (space/privacy)
Waiting times for specialist consultation
Absence of protocols or clear referral guidelines
How adequate do you consider your undergraduate medical training for the diagnosis and management of proctologic conditions? 1 = Totally insufficient 2 = Insufficient 3 = Fair 4 = Adequate 5 = Very adequate
What suggestions would you propose to improve the management of proctologic conditions in primary healthcare? (Optional; free-text response)
Would you be interested or willing to receive additional training to optimize the management of these patients? Yes/No

The survey consisted of 16 items divided into four sections: employment information, clinical experience, diagnostic and referral practices, and perceived barriers and training needs. Closed-ended items included single-response and multiple-response questions. For multiple-response items, including those assessing frequently encountered anorectal conditions and barriers to management or referral, each predefined option was analyzed separately and reported as the number and percentage of respondents selecting it, using the total number of respondents as the denominator. Perception-related items were assessed using 5-point Likert-type response options ranging from 1 (strongly disagree) to 5 (strongly agree), or equivalent anchors according to the content of each item. These response formats were developed specifically for this study and were not derived from previously validated scoring systems. The questionnaire also included one optional free-text item inviting suggestions to improve the management of anorectal conditions in PHC. These responses were reviewed descriptively and used only to provide illustrative examples. No formal qualitative or content analysis, coding framework, or independent coding was performed.

The survey was distributed electronically during August and September 2025. It was sent to each physician’s institutional email address and disseminated through digital channels and the authors’ personal contacts with healthcare centers. Two weekly reminders were sent to encourage participation. Participation was voluntary and anonymous, and the form included an informed consent statement at the beginning. Responses were automatically and anonymously stored in a secure database. The study was approved by the local institutional ethics committee.

Statistical analysis

Categorical variables, including responses to individual 5-point Likert-type items, were summarized using absolute frequencies and percentages. For multiple-response questions, each option was analyzed separately using the total number of respondents as the denominator.

Given that the variables of interest were ordinal and considering the sample size, associations between variables were analyzed using Spearman’s rank correlation coefficient (ρ). Specifically, associations were evaluated between perceived undergraduate training and confidence in performing an anorectal physical examination; between the availability of supplies and examination conditions and clinical confidence; and between the perceived functioning of the referral/consultation system and the feedback received for referred cases.

A p-value < 0.05 was considered statistically significant. Statistical analysis was performed using IBM SPSS Statistics for Windows, Version 30 (Released 2024; IBM Corp., Armonk, New York, United States).

## Results

**Figure 2:**
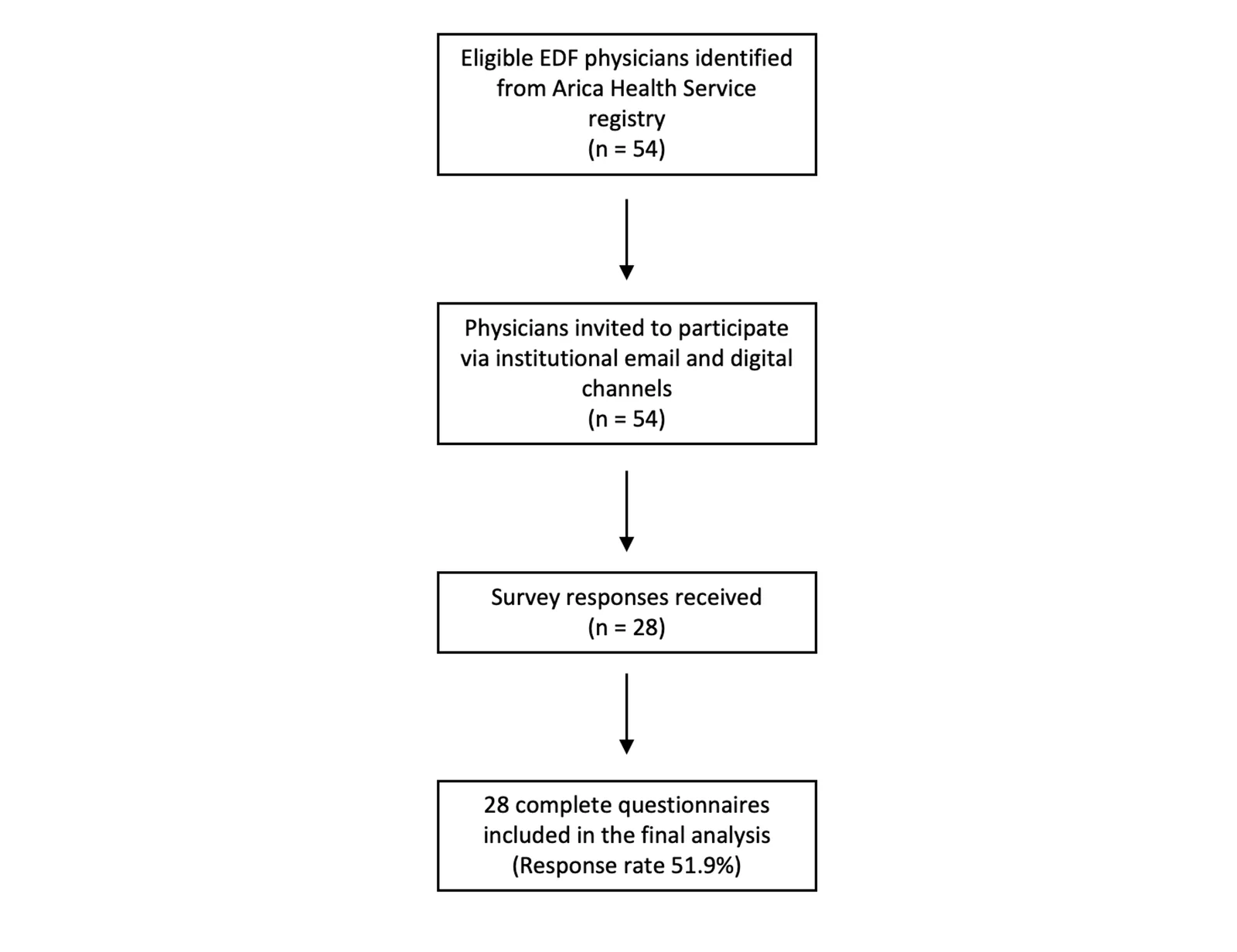
Flow diagram of participant recruitment and inclusion in the study EDF: Destination and Training Stage (Etapa de Destinación y Formación) The image was created by the authors using Microsoft PowerPoint (Microsoft Corp., Redmond, WA, USA).

The survey was sent to a total of 54 EDF physicians from the Arica Health Service. Of these, 28 complete responses were obtained (51.9% response rate) (Figure 2).

Regarding the demographic and occupational characteristics of the sample, most participants worked in Family Health Centers (CESFAM) (82.2%), primarily in the municipality of Arica (82.2%), and had between one and three years of experience in PHC (57.1%) (Table 2).

**Table 2:** Demographic and occupational characteristics of participating physicians (n = 28) Data are presented as absolute frequencies (n) and percentages (%). Variables include municipality of work, type of primary healthcare (PHC) facility, and years of experience working in PHC.

Variable	n (%)
Municipality of work	
Arica	23 (82.2)
Camarones	1 (3.5)
Putre	3 (10.7)
General Lagos	1 (3.5)
Type of healthcare facility	
Family health center	23 (82.2)
Rural health post	5 (17.8)
Years of experience in PHC	
<1 year	6 (21.4)
1-3 years	16 (57.1)
4-6 years	5 (17.8)
>6 years	1 (3.5)

**Figure 3:**
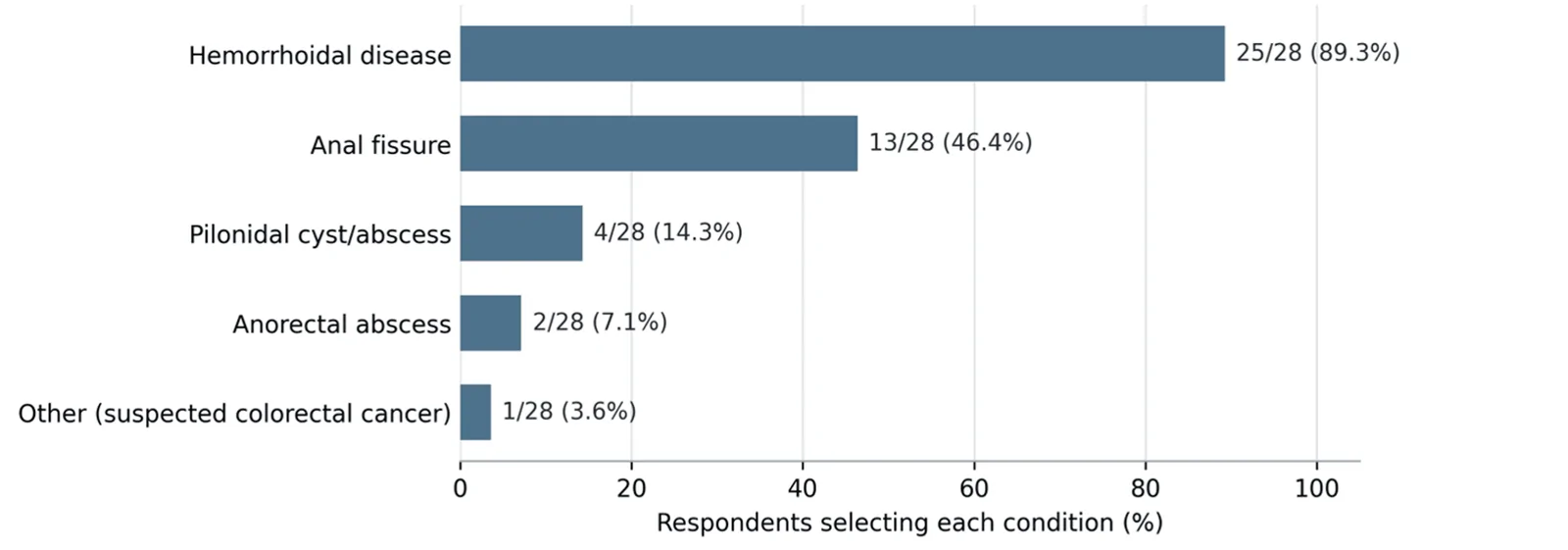
Anorectal conditions reported by EDF physicians as the most frequently encountered in clinical practice Bars represent the number and percentage of respondents selecting each condition (n = 28). Multiple responses were permitted. EDF: Destination and Training Stage (Etapa de Destinación y Formación)

In the clinical experience section, nearly 70% of respondents reported having managed between one and five patients with proctologic complaints in the previous month (Table 3). Hemorrhoidal disease was the condition most frequently reported by respondents (25/28, 89.3%), followed by anal fissure (13/28, 46.4%), pilonidal cyst or abscess (4/28, 14.3%), and anorectal abscess (2/28, 7.1%). One respondent reported suspected colorectal cancer as another frequently encountered condition (1/28, 3.6%) (Figure 3).

**Table 3:** Frequency of medical consultations for anorectal conditions in the previous month Data are presented as absolute frequencies (n) and percentages (%). Categories represent the number of patients with proctologic complaints or anorectal pathology evaluated by each physician during the month preceding the survey.

Patients with anorectal conditions managed in the previous month	n (%)
None	6 (21.4)
1-5	19 (67.9)
6-10	1 (3.6)
>10	2 (7.1)

**Figure 4:**
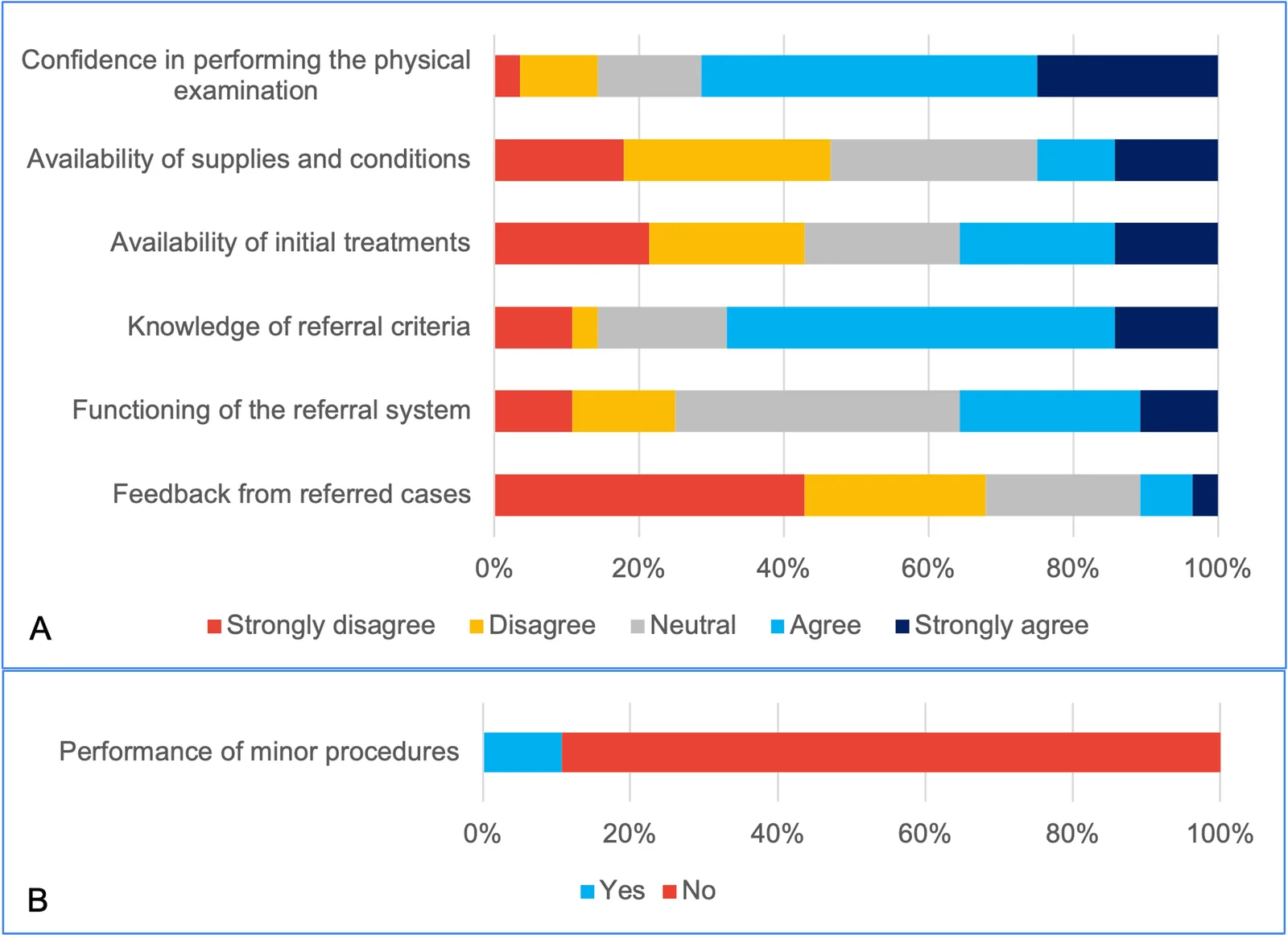
Responses concerning the diagnosis, initial management, and referral of anorectal conditions Distribution of responses concerning diagnosis, initial management, and referral. (A) Responses to five-point Likert-type items, displayed as 100% stacked bars. (B) Performance of minor procedures in PHC, assessed using a binary Yes/No item. Percentages were calculated using all respondents as the denominator (n = 28). PHC: primary healthcare

In the section on diagnosis, initial management, and referral, the highest-rated items were confidence in performing a proctologic physical examination (71.4%) and knowledge of referral criteria to a specialist (67.8%). Conversely, the lowest-rated items were having performed a minor procedure and having received feedback on referred cases (Figure 4).

**Figure 5:**
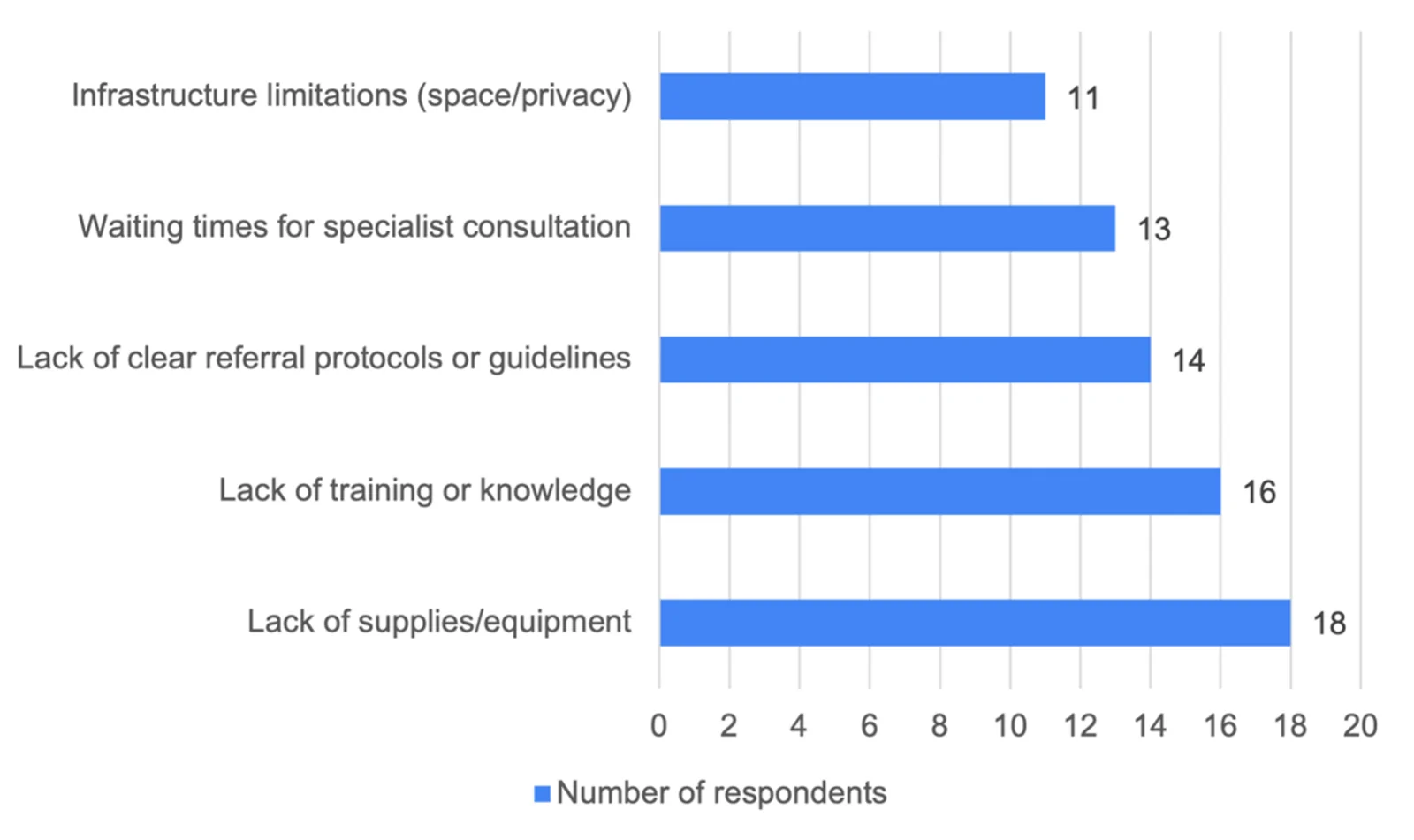
Barriers to the management and referral of anorectal conditions in primary healthcare Bars show the number and percentage of respondents selecting each barrier (n = 28). Multiple responses were permitted.

Regarding barriers and needs identified by respondents, the most frequently reported were lack of supplies/equipment (64.3%), followed by lack of training or knowledge (57.1%) and absence of clear referral protocols (50%). Nearly half of respondents reported prolonged waiting times for specialist evaluation (46.4%), and 39.3% highlighted infrastructure problems or lack of privacy for performing the physical examination (Figure 5). Overall, 71.4% of respondents rated their undergraduate training for the diagnosis and management of these conditions as “adequate” or “very adequate.”

Finally, 12 respondents (42.9%) answered the optional free-text question. Participants’ suggestions to improve the management of these patients primarily focused on clinical training, the development of clear protocols, and improved infrastructure. These were complemented by proposals for specialized teleconsultation and strengthening the problem-solving capacity of PHC through procedure units. A total of 96.4% of respondents indicated that they would be willing to receive some form of additional training.

A moderate positive correlation was observed between perceived undergraduate training and confidence in performing a proctologic physical examination (ρ = 0.563; p = 0.002). No association was found between availability of supplies and confidence in performing the physical examination (ρ = 0.038; p = 0.847). Additionally, perceived functioning of the referral system was significantly associated with feedback received for referred cases (ρ = 0.462; p = 0.013) (Table 4).

**Table 4:** Association between ordinal variables Associations were assessed using Spearman’s rank correlation coefficient (ρ). Data are presented as correlation coefficient (ρ) and p-value. All analyses included the complete sample (n = 28).

Association	Spearman’s ρ	p-value
Undergraduate training ↔ Confidence in performing the proctologic examination	0.563	0.002
Availability of supplies/conditions ↔ Confidence in performing the proctologic examination	0.038	0.847
Functioning of the referral/consultation system ↔ Feedback on referred cases	0.462	0.013

## Discussion

Anorectal disorders represent a frequent reason for consultation in PHC. This group encompasses a wide spectrum of conditions ranging from hemorrhoidal disease to malignant lesions. Although no studies have comprehensively grouped all conditions within this category, their prevalence is estimated at 10-15% in the general population, with up to 80% of symptoms potentially attributable to underlying anorectal pathology [6]. Associated consequences include chronic anal pain, bleeding, and constipation, which may progress to severe complications such as perianal sepsis and fecal incontinence [7].

PHC constitutes the entry point to the health system, and consultations for these conditions are often delayed, largely due to the anatomical location of symptoms [8]. Accurate diagnosis is essential for appropriate management and timely referral. However, diagnostic inaccuracy in anorectal conditions ranges in the literature from 17% to over 50% [4,9,10], including among non-surgical specialists [11]. Misdiagnosis and delays in appropriate referral may contribute to complications and persistence of symptoms, with a substantial negative impact on patients’ quality of life [10,12].

The value of the physical examination lies in its ability to improve diagnostic accuracy and to distinguish between conditions that frequently present with overlapping symptoms. A Spanish study involving PHC physicians found that anal inspection was omitted in more than 40% of cases and digital rectal examination in more than 80%, significantly compromising diagnostic precision [13]. Grucela et al. [4], in a prospective study, reported diagnostic accuracy below 50% across most specialties, except for surgeons and emergency physicians, and performance did not improve with years of clinical experience. Similarly, Goldstein [10] observed a 51% diagnostic accuracy rate among PHC physicians, with a 25% delay in referral and a 60% rate of complications associated with such delays.

Another major implication of diagnostic inaccuracy is the misclassification of early anal or rectal cancer as benign anorectal disease. In a retrospective study of patients with anal cancer, Bingmer et al. [14] found that 57% were initially diagnosed with benign anorectal conditions, and 25% of these patients had experienced symptoms for more than six months prior to correct diagnosis.

The present study describes the perceptions, barriers, and experiences of EDF physicians in the Arica and Parinacota Region regarding the management and referral of anorectal disorders in PHC. Most respondents reported encountering anorectal conditions during the preceding month, with hemorrhoidal disease and anal fissure being the most commonly selected conditions. This pattern is consistent with national and international reports identifying benign anorectal diseases as common reasons for consultation and referral in primary care [4,6,7].

One of the most relevant findings was the moderate positive correlation between perceived undergraduate training and confidence in performing a proctologic physical examination. Proctology training during medical school is often limited and characterized by minimal practical exposure, which may lead to lower rates of proctologic physical examination and a greater tendency toward early referral. This aligns with evidence demonstrating low self-confidence in performing and interpreting digital rectal examination findings among medical students [15], underscoring the importance of undergraduate training in adequately preparing newly graduated and EDF physicians to manage these patients.

Furthermore, receipt of feedback from secondary care was positively associated with physicians’ perceptions of referral-system functioning, underscoring the close relationship between communication and perceived coordination across levels of care. In the context of surgical conditions, counter-referral may serve not only a clinical function but also an educational one by providing PHC physicians with information about diagnostic decisions and subsequent management. Poor communication between specialists and PHC physicians, with limited information regarding processes and care provided at the secondary level, has been shown to adversely affect continuity and quality of care [16].

The most frequently reported barriers - including lack of supplies, knowledge deficits, absence of defined protocols, and prolonged waiting times - are consistent with limitations described in studies from other clinical areas in northern Chile [17]. Internationally, significant discrepancies have been observed between PHC management of anorectal disorders and established clinical guidelines [18], likely related to the absence of clear and accessible protocols. Notably, the high willingness to undergo additional training observed in this study represents a tangible opportunity to design and implement educational interventions aimed at strengthening diagnostic and management competencies in PHC.

Regarding strategies to improve diagnostic accuracy, Spanos et al. [19] demonstrated a significant increase in diagnostic precision for anorectal conditions - from 43.1% to 80.6% (p < 0.05) - after incorporating three supervised outpatient coloproctology rotation sessions for final-year medical students. A recent review highlights substantial gaps in clinical training in this field and emphasizes the need for structured educational programs incorporating audiovisual materials and three-dimensional simulation models to enhance knowledge retention and clinical application, with the greatest benefit observed among less experienced practitioners [20]. Such approaches may be particularly applicable to EDF physicians and early surgical trainees. These recommendations support our findings, in which confidence in proctologic examination was associated with perceived training, and reinforce the plausibility of targeted educational interventions to enhance PHC problem-solving capacity.

These findings are particularly relevant in geographically remote regions of the country, where timely access to specialist care is limited, and PHC plays a central role in containing specialist demand. Strengthening the initial management of benign anorectal disease may improve quality of care, reduce unnecessary referrals, and optimize resource utilization within the healthcare network.

This study has several limitations. First, the relatively small sample size, restriction to a single geographic region, and response rate of 51.9% may limit the generalizability of the findings to other PHC settings. Nonresponse and self-selection bias cannot be excluded, as physicians with greater interest in anorectal conditions, stronger perceptions of referral barriers, or greater willingness to receive additional training may have been more likely to participate. Second, the questionnaire was developed specifically for this study and did not undergo formal expert review or psychometric evaluation of its validity and reliability. Although it was preliminarily assessed by two PHC physicians for clarity, comprehensibility, and feasibility, this process does not establish that the instrument measures the constructs of interest accurately and reproducibly. Third, the study relied on self-reported perceptions and experiences, which may be affected by recall bias, social desirability bias, and common-method variance. In particular, perceived referral-system functioning and receipt of feedback were assessed using similar Likert-type items and represent conceptually related constructs; therefore, construct overlap may have contributed to the observed correlation. Finally, the cross-sectional design and simultaneous measurement of the variables preclude establishing directionality or causal relationships between perceived training, clinical confidence, feedback, and referral-system functioning.

Future studies should include larger, multicenter samples involving different regions and healthcare systems to evaluate the generalizability of these findings. Prospective studies assessing the impact of structured educational interventions, clinical training programs, and referral pathway improvements on diagnostic accuracy, management confidence, and referral appropriateness would be valuable. Additionally, future research should consider the development and validation of standardized instruments to objectively evaluate competencies and barriers in the management of anorectal disorders among PHC physicians.

## Conclusions

Most EDF physicians working in PHC in the Arica and Parinacota Region reported encountering anorectal conditions, with moderate clinical confidence and barriers that are primarily educational and related to healthcare network organization. Perceived adequacy of undergraduate training was associated with confidence in performing the physical examination, while reported feedback from secondary care was associated with perceived referral-system functioning. These findings support the implementation of targeted educational strategies to improve diagnostic accuracy, along with strengthened coordination between PHC and specialist care, with the aim of improving system efficiency and problem-solving capacity within PHC.
